# Copper–Calcium Hydroxide and Permanent Electrophoretic Current for Treatment of Apical Periodontitis

**DOI:** 10.3390/ma14030678

**Published:** 2021-02-02

**Authors:** Agron Meto, Etleva Droboniku, Elisabetta Blasi, Bruna Colombari, Emiljano Tragaj, Gabriele Cervino, Luca Fiorillo, Aida Meto

**Affiliations:** 1 Department of Therapy, Faculty of Dentistry, University of Aldent, 1000 Tirana, Albania; agronmeto@yahoo.com (A.M.); emiljanotragaj@yahoo.com (E.T.); 2 Department of Dental Therapy, Faculty of Dental Medicine, University of Medicine, 1005 Tirana, Albania; etleva.droboniku@umed.edu.al; 3 Department of Surgical, Medical, Dental and Morphological Sciences with Interest in Transplant, Oncological and Regenerative Medicine, University of Modena and Reggio Emilia, 41125 Modena, Italy; elisabetta.blasi@unimore.it (E.B.); bruna.colombari@unimore.it (B.C.); 4 Department of Biomedical and Dental Sciences, Morphological and Functional Images, University of Messina, 98100 Messina, Italy; gcervino@unime.it; 5 Multidisciplinary Department of Medical-Surgical and Odontostomatological Specialties, University of Campania “Luigi Vanvitelli”, 80121 Naples, Italy

**Keywords:** apical periodontitis, destructive focal sizes, copper–calcium hydroxide, electrophoretic current, root canal, endodontic regeneration, histology, tooth discoloration

## Abstract

Endodontic failure has been and continues to be a problem for endodontics-specialists. Complicated anatomy, numerous foramens, and accessory canals are an environment for microorganisms to infect the teeth. The purpose of the present work was to evaluate the regeneration of copper–calcium hydroxide (Cupral)-endodontically treated teeth diagnosed with apical periodontitis using an electrophoresis technique. In total, 132 patients, aging from 19 to 65 years old, underwent endodontic treatment mono- and multi-radicular teeth, with complicated canals from January 2019 to June 2020. The patients were divided into two groups: (i) the control group—which included 54 patients (*n* = 62 teeth) receiving endodontic paste (Calcipast + 1) and, as final filling, the AH-Plus^TM^ cement—and (ii) the Cupral group, which included 78 patients (*n* = 80 teeth) receiving Cupral paste plus the electrophoretic current and, as final filling, the Atacamit-alkaline cement. The clinical cases were periodically observed along an 18-month follow-up period via radiography. Data were expressed as focal size of the lesions (mean ± standard error (SEM) of all the radiographic outcomes) observed in each group at each interval point. Statistical analysis was performed using the Student’s *t*-test that allowed us to compare the control and Cupral groups; the statistical significance was set at *p* < 0.05 and *p* < 0.01, where the latter was highly significant. Before treatments, the focal sizes were 4.8 mm and 4.95 mm for control and Cupral-treated groups, respectively. After 6 months, the mean focal sizes were 3.9 mm and 2.14 mm for the control and Cupral groups, respectively. After 12 months, in the control group, the mean focal size was measured at 2.8 mm, while, in Cupral group, the lesion size decreased down to 0.31 mm and a highly dynamic regeneration of the destructive focal-bone occurred. After 18 months, the lesions were further significantly reduced in the control group (mean values of 2.62 mm), while they were barely detectable in the Cupral group (0.2 mm). In conclusion, we provide initial evidence that the Cupral-electrophoresis methodology is effective in treating destructive periodontitis of teeth with problematic canals up to 18 months, thus allowing teeth preservation.

## 1. Introduction

The cause of low effectiveness in endodontic teeth treatments is not only the complicated system of root canals but also the porous structure of root dentin [1]. There are up to 60,000 dentinal tubules/mm^2^ with an average diameter of 800 nm where microorganisms persist [2]. Non-vital teeth can be infected at any moment by secondary microorganisms from the periapical region or the mouth cavity. In this way, it can lose the defense immune factor and supplying the pulp with blood [3]. Apical periodontitis is also found in immature teeth, which require regeneration of the pulp tissue to survive. One of the great problems with an immature necrotic tooth is to have an effective disinfection of the canal in order to allow successful regenerative endodontic treatment, as also confirmed by radiological and histological data [4,5,6]. The presence of a large number of lateral canals, complicated anatomical morphology, as well as numerous foramens, lead to features opening to microorganisms localization and persistence also as biofilm [7]. In most of the cases, periapical inflammatory processes penetrate inside the tooth without symptoms [8]. In traditional endodontics for canal-disinfection, active irrigants are necessary to penetrate the endodontic cavity and peripheral parts of root canal system; their effects are reduced when encountering pulp and dentin components [9,10,11]. Nowadays, several data have demonstrated that different components in dentin may affect the antibacterial properties of compounds such as sodium hypochlorite (NaOCl), chlorhexidine digluconate (CHX), iodine potassium iodide (IPI), and Tetraclean [12,13,14]. Indeed, it is known that the interaction of chlorine with host proteins leads to necrotic tissue melting, but, on the other hand, bactericidal activity is lost [15]. In addition, under the action of NaOCl, the dentin becomes smoother and easily fractured [15]. A group of authors tested the in vivo efficacy of 2% CHX gel and root filling with Resilon/Epiphany system to inhibit the development of apical periodontitis [16].

The bactericidal activity of calcium hydroxide (Ca(OH)_2_) in the root canal weakens quickly because of the oxidation reaction which occurs when Ca(OH)_2_ comes in contact with pulp proteins forming calcium proteinate [17]. The paste limits the growth of endodontic bacteria, but does not completely abrogate it. Thus, microorganisms can persist in the canal system and lead to apical periodontitis, which can often not be visible upon the radiologic examination but can be detected by ex vivo histological studies [18,19]. Currently, despite improvement in endodontic techniques, the percentage of failure still remains high, likely also because of the ability of certain endodontic pathogens to locally produce a robust biofilm that allows their intra-radicular persistence [20,21].

Over the last few years, a copper–calcium hydroxide (Cupral, Humanchemie GmbH, Alfeld, Germany) compound has become widely available for its potent antimicrobial properties [22,23,24,25] in pulp pathologies-treatments. The Cupral-based treatment was developed by Prof. Adolph Knappwost, Universities of Hamburg and Tübingen, Germany. Cupral has numerous physico-chemical characteristics that allow it to be used in association with an electrophoresis technique, which solves the task that lies before endodontics specialists [23,24,25]. Such a technique has been shown to be active not only against bacterial and fungal vegetative cells but also against spores and viruses; its wide-spectrum efficacy renders this approach highly interesting [23,24,26].

We based this study on the American Association of Endodontists (AAE) about the standardization of diagnostic terms [27].

The purpose of the present work was to radiographically evaluate the regeneration of teeth, diagnosed with apical periodontitis and treated with Cupral and electrophoresis and then checked during an 18-month follow-up period.

## 2. Materials and Methods

### 2.1. Patients’ Selection

**Scheme 1:**
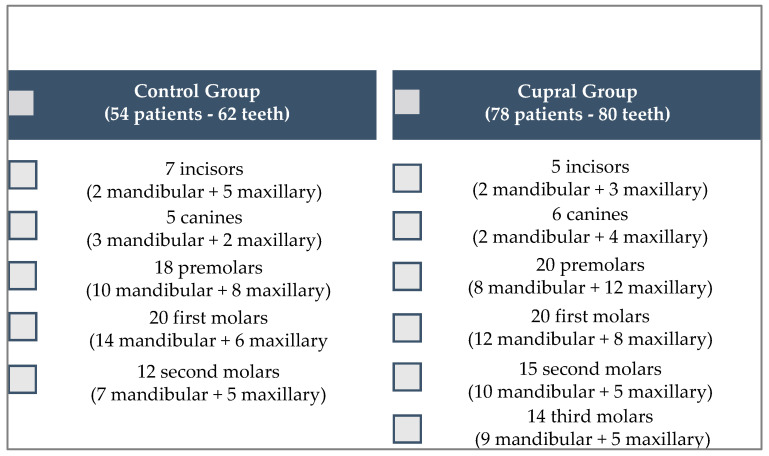
Groups division of treated teeth.

A total of 132 patients (aged 19–65 years old) with apical pathologies were treated in from January 2019 to June 2020. The patients were evaluated based on their complaints and presence in the follow-up examinations. They provided their consent after being informed at the beginning of the procedures, about the possible pains and side effects, such as crown discoloration during treatment. Approval of the study was obtained from the Local Ethics Committee (Protocol no. 1223/2019). In all the patients, apical periodontitis was associated with mono- and multi-radicular lesions; accentuated curves and complicated canals (obliterations, hypercementosis) were also detected, according to Estrela 2008 [28]. Patients were divided into control and Cupral groups and observed in an 18-month follow-up period. The patients’ teeth were selected and arranged as shown in the flow-chart (Scheme 1).

### 2.2. Criteria of Inclusion and Exclusion of the Cases

No age restriction criteria were set in either group. The inclusion criteria considered were as follows: all cases with pulp pathology due to carious complications; all cases that needed re-treatment and that showed changes in the apical periodontal space; cases with obliterated and curved canals accompanied by periapical destruction. The exclusion criteria considered were as follows: patients with cardiovascular disease with pacemaker, pregnant women, and patients allergic to copper or other metals. The groups were screened by clinical and radiographic examinations before deciding the endodontic methodology to use. Patients were divided into 2 groups, in that of Control and Cupral one, as detailed in the sub-sections below (Section 2.3 and Section 2.4).

### 2.3. Endodontic Traditional Treatment of Control Group

After the diagnosis, the endodontic protocol was applied, starting with the rubber dam isolation. As next step, the cavity access was performed with the cavity access set burs (Dentsply Maillefer, Ballaigues, Switzerland). The chemo-mechanical preparations for each tooth started with the inspection of canal entrances using manual Ni-Ti files sizes 10–15 (Dentsply Maillefer, Ballaigues, Switzerland) in alternation with ProTaper Universal Hand Shaping Files SX-S1-S2 (Dentsply Maillefer, Ballaigues, Switzerland). Then, for the root canal finishing using ProTaper Universal F1-F2-F3 (Dentsply Maillefer, Ballaigues, Switzerland), were used. The coronal part of the root was enlarged through Gates-Glidden drills sizes 1–2, (Dentsply Maillefer, Ballaigues, Switzerland).

Canal preparation was completed using the step-back technique. After each file, the irrigation with 5.25% NaOCl (Cerkamed, Stalowa Wola, Poland) was performed, followed by 17% ethylenediaminetetraacetic acid (EDTA; Cerkamed, Stalowa Wola, Poland) for smear layer removal. After this step, physiological saline solution and then 2% CHX (GLUCO-CHEX; Cerkamed, Stalowa Wola, Poland) were applied using a NaviTip 30-G needle (Ultradent, South Jordan, UT, USA) with passive irrigation. Each canal was dried using sterile paper points (Meta Biomed, Colmar, PA, USA). After that the intracanal medication with Ca(OH)_2_ compound with iodoform (Calcipast + 1; Cerkamed, Stalowa Wola, Poland) was inserted inside the canal and refreshed twice every 2 weeks. The cavities were closed provisionally with a sterilized cotton pellet and Cavit^TM^ (3M, Neuss, Germany). After the second session, Calcipast + 1 was cleaned from the canals using physiological saline solution. Each canal was dried using sterile paper points (Meta Biomed) and the canal filling was done through AH-Plus^TM^ (Dentsply Maillefer, Ballaigues, Switzerland) cement, using lateral condensation technique with gutta-percha points (Meta Biomed, Colmar, PA, USA). The final tooth crowns filling were completed with the help of Competence Universal Composite^®^ (WP GmbH, Barmstedt, Germany).

### 2.4. Electrophoresis-Based Endodontic Treatment in Cupral Group

A commercially available copper–calcium hydroxide—based (Cupral, Humanchemie GmbH, Alfeld, Germany) compound, containing calcium hydroxide (Ca(OH)_2_)-highly dispersed, copper sulphate (II) (CuSO_4_), calcium sulphate (CaSO_4_), copper hydroxide (II) (Cu(OH)_2_), methylcellulose [C_6_H_7_O_2_(OH)_x_(OCH_3_)_y_]_n_, and distilled water (dist. H_2_O) was used. In the Cupral-treated group, we followed the minimally invasive protocol, recommended by Knappwost [23]. After the diagnosis, the isolation with the rubber dam was performed. As a next step, the cavity access was performed with the cavity access set burs (Dentsply Maillefer, Ballaigues, Switzerland).

Here, we performed the crown-down technique up to 1/3 and 2/3 (in cases with obliterated canals) of the canal length according to the presented endodontic cases. The coronal part of the root was enlarged through using Gates-Glidden drills sizes 1-2-3 (Dentsply Maillefer, Ballaigues, Switzerland) and was then mechanically processed with K-file sizes 40–15 (Dentsply Maillefer, Ballaigues, Switzerland). The canals were irrigated with dist. H_2_O after each file, thus avoiding NaOCl, EDTA or CHX usage in order to not compromise the effects of Cupral paste.

Before electrophoresis session, each canal was dried using sterile paper points (Meta Biomed, Colmar, PA, USA), then a portion (size of 2–3 mm) of Cupral paste was taken and placed on a sterile glass plate and mixed it with dist. H_2_O to a creamy consistency. The paste was prepared thinner for mandibular canals, while for those maxillary was slightly thicker. The consistency was introduced into the canal by the help of lentulo spiral fillers size 1 (Dentsply Maillefer, Ballaigues, Switzerland). For both maxillary and mandibular distal teeth, Cupral paste was used; in the frontal teeth, a portion of Cupral and 9 portions of the Ca(OH)_2_-highly dispersed paste (Humanchemie GmbH, Alfeld, Germany) were applied at a ratio 1:9. This combination-paste was due to the color effect of Cupral and also this paste was inserted into the root canal by the lentulo spiral fillers size 1 (Dentsply Maillefer, Ballaigues, Switzerland). After the paste was placed within the canal, the needle of cathode (K^−^) was inserted inside the endodontic cavity and anode (A^+^) clamp (placed in the internal site of the inferior lip); the current was applied by switching the apparatus COMFORT II (Humanchemie GmbH, Alfeld, Germany). 

**Scheme 2:**
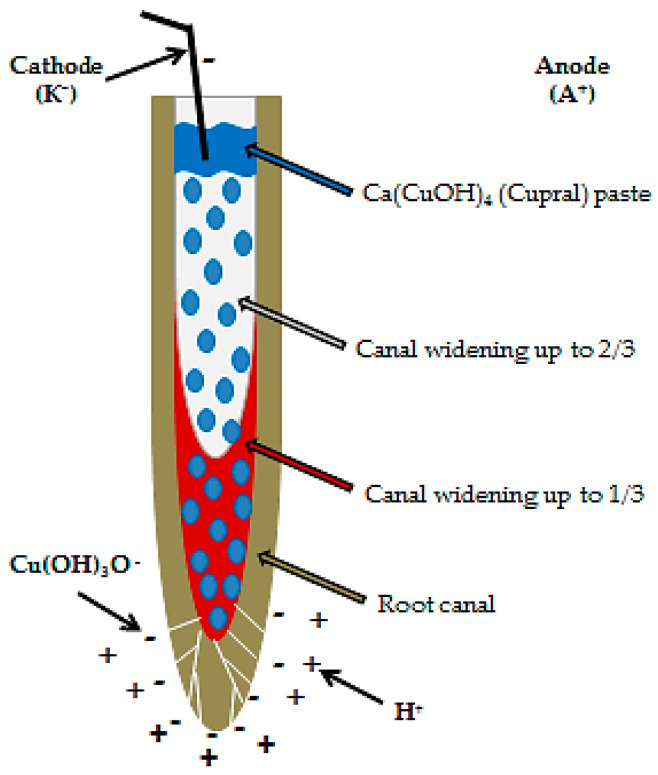
Possible mechanism of ion electro-transmission in the radicular canal during electrophoresis in the presence of Cupral. This technique is based on the principles of electrophoresis and ionophoresis, where ions and negative-load particles move to the positive ones. The electric current only helps to transfer the particles of Cupral’s ions inside the root canal and ramifications. The transport of this quantity happens according to Faraday’s law, where the past quantity of electric current, i.e., the amount of permanent electricity expressed by the creation of increased current in time (mA × min), when the procedure takes place. The amount mA × min corresponds to the dose determination of Cupral inserted into the destructed focal site.

The first electrophoretic session was completed with 7.5 mA·min·canal^−1^, depending on the anatomic construction-canal (see, Scheme 2).

After the electrophoresis procedure, the cavities were closed provisionally with a sterilized cotton pellet and Cavit^TM^ (3M, Neuss, Germany) until the second session application. The latter was performed after 8 days, removing the provisional filling (Cavit^TM^, 3M) and refreshing the canals by rinsing with dist. H_2_O, drying with sterile paper points (Meta Biomed) and a new portion of Cupral paste was restored with lentulo spiral filler size 1 (Dentsply Maillefer), thus creating a suspension of Cupral and followed the same technique as the first session for distal and frontal teeth (in combination with Ca(OH)_2_-highly dispersed paste (Humanchemie GmbH), performing in each canal 7.5 mA × min/canal.

After the second electrophoretic session, the treated canals were rinsed with dist. H_2_O, dried with sterile paper points (Meta Biomed, Colmar, PA, USA), and finally filled by means of Atacamit (Humanchemie GmbH, Alfeld, Germany)-alkaline cement (owning permanent sterilization effects) and gutta-percha points (Meta Biomed, Colmar, PA, USA), using the lateral condensation technique. The final teeth crowns filling were completed with the help of a Competence Universal Composite^®^ (WP GmbH, Barmstedt, Germany).

The follow-up for both groups was performed obtaining intraoral radiographic images before and after the final canals filling, after 6, 12 and 18 months. The parallelism technique associated with the Rinn centerers was performed for both periapical and bitewing radiographs. In addition, appropriate devices were used, such as receptor instruments with ring guides, cotton rolls, standard bite blocks, and bitewing tabs. The digital receptor was positioned vertically or horizontally parallel to the radiographed teeth. The X-ray beam was directed at right angles to the teeth and the receptor.

### 2.5. Measurement of Apical Bone-Destruction

Measurements of the focal sizes were obtained analyzing the radiographic data of both groups, considering the apical bone destruction. This was done with the help of a ruler (Supplementary Material, Figure S1) relying on the International System of Units, which is used for scientific and daily purposes [29]; this allowed us to evaluate the apical lesions and express their entity in millimeters (mm).

### 2.6. Histological Evaluation

A few Cupral-treated cases (3 teeth in total) during the 18 month-follow up showed a fracture, which was likely due to the functional overload and we were obligated to extract them. After the extraction, these roots were inserted into a labeled container with 10% neutral buffered formalin (Formaldehyde 10% *v*/*v* buffered 4% *w*/*v* for histology, Titolchimica S.p.a, Pontecchio Polesine, Italy) for fixation and then delivered to the Histology Laboratory for histological examination. Then, they were resected and placed in 37% formaldehyde (Fisher Scientific, Fair Lawn, NY, USA). After removal of all soft and hard tissues from the samples, they were next placed in 14% EDTA (decalcifier for analysis histological, Titolchimica S.p.a, Pontecchio Polesine, Italy) for the decalcification process for 6 days, changing every day the solution. Additionally, to remove the decalcification solution, the samples were rinsed under a running tap water wash for 20 min following the dehydration process by immersion in 96 °C ethyl alcohol. The samples were then embedded in Paraplast paraffin (Fisher Scientific, Fair Lawn, NY, USA) blocks at 58 °C. After this, the blocks were cut using the Leica microtome (RM2125 RTS, Leica Mikrosysteme Vertrieb GmbH, Wetzlar, Germany) to a thickness of 5 μm. The cuts were processed longitudinally and horizontally. The slides were stained with the Hematoxylin and Eosin (H&E) and then evaluated by the light microscope Nikon Eclipse E200 (Nikon Instruments, Melville, NY, USA) with 400× of magnification.

### 2.7. Statistical Analysis

Data were expressed as mean ± standard error (SEM) of all the focal sizes outcomes obtained. Statistical analysis was performed using the Student’s *t*-test that allowed comparing Control vs. Cupral groups. Values of *p* < 0.05 and *p* < 0.01 were considered as statistically significant and highly statistically significant, respectively. 

## 3. Results

### 3.1. Radiographs of Clinical Cases Treated with the Traditional Methodology

**Figure 1:**
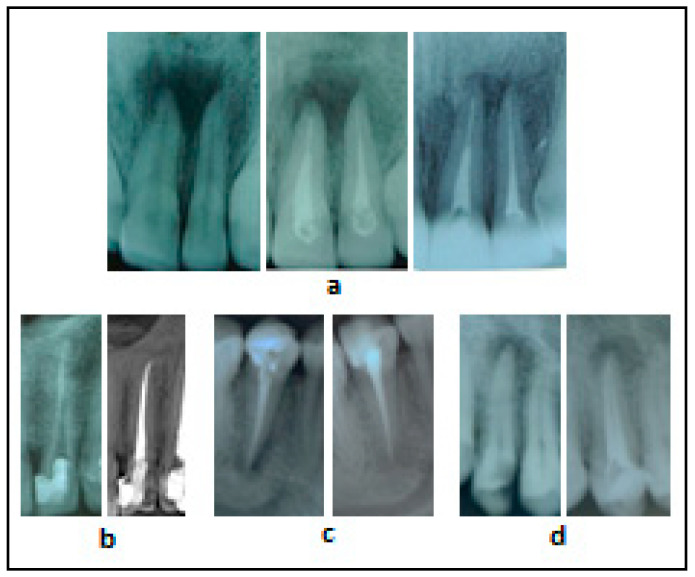
Radiographs of apical periodontitis cases from the control group. (**a**) female, 55-years old, teeth no. 21 and 22 (before treatment, after 6 and 18 months); (**b**) male, 40-years old, tooth no. 22 (before treatment and after 18 months); (**c**) female, 45-years old, tooth no. 45 (before treatment and after 18 months); (**d**) male, 19-years old, tooth no. 24 (before treatment and after 12 months).

In the radiographs presented in Figure 1, a decrease in the destructive focal sites in the periapex was observed in the cases of control group, after treatment with Calcipast +1 and the final filling with AH-Plus^TM^ cement.

An interesting fact is that X-rays observed a total disappearance of the apical infection, which could not be observed in all the cases. However, clinically the patients claimed to have no symptoms. In the periapex, there were radiolucency areas, which indicate that the regeneration is not over yet after 12 and 18 months but it continues at a slow rate (note: some of the cases are still under our observation).

### 3.2. Radiographs of Clinical Cases Treated with Cupral Associated with Electrophoresis

**Figure 2:**
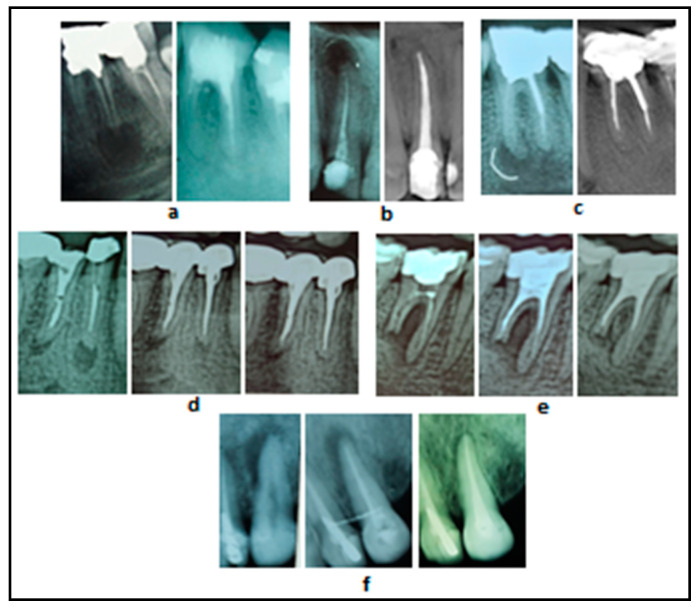
Radiographs of apical periodontitis cases from the Cupral group. (**a**) Female, 25-years old, tooth no. 36 (before treatment and after 12 months); (**b**) male, 51-years old, tooth no. 12 (before treatment and after 12 months); (**c**) male, 45-years old, tooth no. 36 (before treatment and after 18 months); (**d**) female, 42-years old, teeth no. 44 and 45 (before treatment, after 12 and 18 months); (**e**) male, 23-years old, tooth no. 46 (before treatment, after 6 and 12 months); (**f**) female, 26-years old, tooth no. 23 (before treatment, after 6 and 18 months).

In all cases treated with Cupral and electrophoresis, a massive reduction of the clinical lesion was observed over time. After 12 and 18 months, a dense area around the periapex was seen, which indicates the complete regeneration of the destructed apical structure, as shown in Figure 2.

### 3.3. Clinical Outcomes with Respect to Patients’ Age 

In Table 1, the clinical outcome was expressed stratifying the patients according to their age. In both groups, the percent of endodontic success partially decreased by the age. In any case, the Cupral group consistently provided slightly better results than the control group.

### 3.4. Measurements of the Focal Sizes

**Figure 3:**
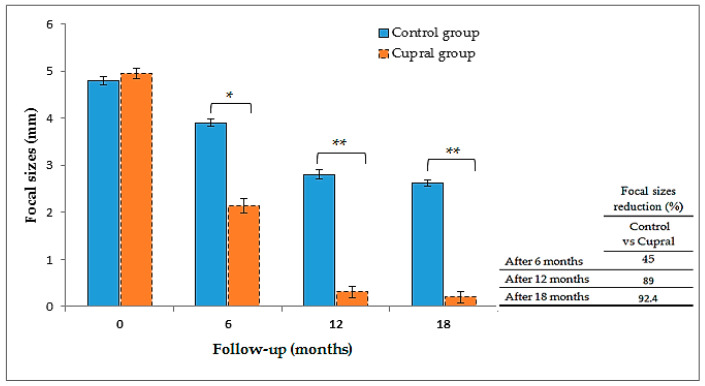
Measurements of focal sizes and drastic reduction upon Cupral-electrophoresis treatment. The focal sizes values (mean ± SEM of focal measures in mm) are represented by the columns at time 0 and during the follow-up in months. The focal sizes reduction (%) related to each condition is shown in the right insert. Statistical analysis was performed using the Student’s *t*-test; * *p* < 0.05, ** *p* < 0.01: control (traditional) vs. Cupral groups.

The focal areas, visible in the radiographs, were measured using a ruler and expressed in mm, before treatment (time 0) and after 6, 12, and 18 months (Figure 3).

At time 0, the focal sizes were 4.8 mm and 4.95 mm for control- and Cupral-treated groups, respectively. After 6 months of observation, the mean focal sizes were 3.9 mm and 2.14 mm for control- and Cupral-treated groups, respectively. These data indicated that Cupral treatment was significantly more effective than the traditional treatment in reducing focal sizes down to about 45% after 6 months. After 12 months, in the control group, the mean focal sizes measured of 2.8 mm; in Cupral-treated group, a highly dynamic regeneration of destructive focal-bone occurred, since we observed a decrease of the lesion to 0.31 mm. Once again, Cupral treatment provided the best results, with a drastic reduction of 89% of focal sizes. After 18 months of treatment, the damaged bone structures were further significantly reduced in the control group, with lesions showing mean values around 2.62 mm; meanwhile, in the Cupral-treated group, the regeneration beyond apex reached 0.2 mm, with a focal area reduction of 92.4% respective to the control group.

### 3.5. Histological Analysis of the Extracted Teeth

**Figure 4:**
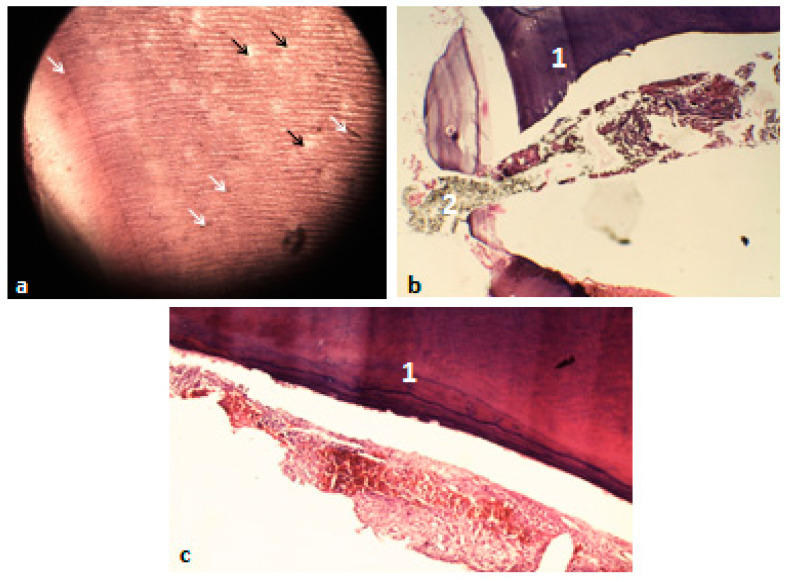
Histologic microphotographs of extracted teeth (root treated in two sessions with Cupral). The samples were processed, stained with Hematoxylin and Eosin (H&E) and cut in longitudinal section. (**a**) There is an impregnation of the dentinal canals with copper crystals along their entire length, highlighted in light brown color (white arrows). The free, unimpressed intracanal matrix is seen in white color (black arrows). (**b**) Apical section: Intensive impregnation with copper sulfite of the endodontic canal wall, where a homogeneous penetration is observed. Copper crystals in the dentinal tubules along the entire length of the root canal walls are also evident (1). Copper hydroxide precipitation at the root apex highlighted by the dark green color (2). (**c**) The canal wall is intensely impregnated with copper sulfite; a massive impregnation of the dentinal canals is also observed (1). Representative images are shown in a magnification of 400×.

**Figure 5:**
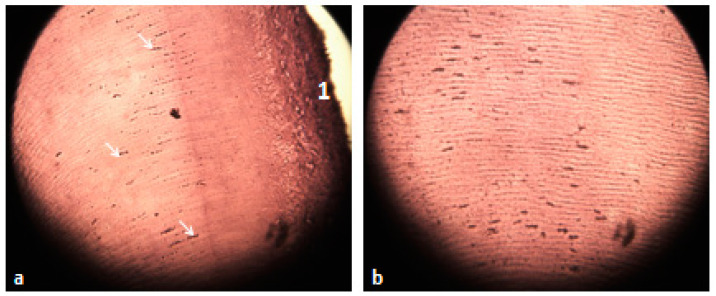
Histologic microphotographs of extracted teeth (root treated in two sessions with Cupral). The samples were processed, stained with H&E and cut in horizontal section. (**a**) The inner wall of the canal dentin has an intense impregnation with copper sulfite (1); also the penetration of copper crystals along the entire length of the dentinal tubules is observed (white arrows). (**b**) The extended penetration of copper crystals in the dentinal tubules is quite noticeable, thus ensuring permanent sterility. Representative images are shown in a magnification of 400×.

Figure 4 and Figure 5 show the histologic images of extracted Cupral-treated teeth. The histological analysis showed the penetration of copper hydroxide into the radicular canal and dentinal tubules.

## 4. Discussion

In this study, we described a novel method as a successful and safe alternative for endo-canalar treatment. The preparation and treatment techniques in the present work were different, because we wanted to emphasize which one improved the outcome in the treatment of apical periodontitis. The technique used in control group, the traditional method, was related to the mechanical and chemical preparation up to the physiological narrowing in cases with passable root canals; while in the Cupral group, the technique of mechanical processing of the canals was different, because the canal was prepared up to 2/3 of its length, or less as far as the complicated anatomy of the tooth root allowed. Here, we performed a mini-invasive treatment where the canals were expanded minimally and part of them up to 1/3 of the length remained untreated, which was sterilized by the electric current of copper hydroxide ions. A long-term sterility of the canals was obtained through impregnation with copper sulfite and as demonstrated by the abrogation of the destructive apical lesions, stimulation of the osteogenesis was obtained through hydroxyl ions.

In the follow-up of the control group, a positive result was found, where we detected 4.8 mm prior to treatment, then 3.9 mm after 6 months, and 2.8 mm and 2.62 mm after 12 and 18 months, respectively. Interestingly, in the Cupral-treated group, a gradual and consistent improvement of the regeneration has been observed, where from 4.95 mm prior to treatment to 2.14 mm after 6 months, to 0.31 mm after 12 months and 0.2 mm after 18 months. The results obtained showed that the Cupral-electrophoresis technique was more effective in the treatment of cases with apical periodontitis compared to traditional ones. The healing process of inflammatory focal in various age groups progressed at different speeds in both groups. As can be predicted, younger ages (19–35 years old) had the maximum regeneration rate, with elder ages in both groups having a lesser regeneration rate. The minimal regeneration occurred in old patients over 51 years of age (85 vs. 90% for control and Cupral groups respectively, as shown in Table 1). We think that, among many factors that influence the results of apical periodontitis treatment, the immune system, as well as the patient’s organism resistance, are determining factors.

The clinical and radiological follow-up of the patients showed in control group that the traditional method solved the pain but the apical regeneration was slow and unsure. In line with our results, the literature claims that the bactericidal activity of Ca(OH)_2_ in the canal decreases rapidly, when in contact with the albumin of the root pulp forming calcium proteinate. In addition, the activity of the compounds is known to also decline by the soaping reaction. Nowadays, several studies have proven that different components in dentin are the cause of phenomena that inhibit the antibacterial activity of Ca(OH)_2_, CHX, etc. [30]. According to this and other data [31], bacteria have been detected in the root environment after 4 weeks of treatment with Ca(OH)_2_, regardless of the method of condensation of the latter in the root canal. Accordingly, Peters et al. [32] stated that, despite the canals preparation and filling with Ca(OH)_2_ paste, the bacterial chance to infect root canals is high in the period between treatment sessions.

The traditional Ca(OH)_2_ paste limits endodontic bacteria growth, but does not enable their complete elimination [7]. With time, canals may be contaminated once again, by microorganisms that infiltrate from the oral cavity or from the periapical region through multiple foramina within lateral canals [3,7]. In more than 60% of endodontic failures, it has been possible to reinforce the view that, nowadays, traditional procedures, in principle, do not provide a permanent solution for root canal infection fighting [33]. Despite the wide arsenal of modern tools used in clinical practice, antimicrobial processing of root canals is not always effective; in 15–30% of cases, the development of inflammatory processes in periapical tissue is observed [34]. Endodontic pathogens deeply penetrate into the accessory canals and into the porous structure of the canal dentin; as a consequence, they damage the periodontal connective tissue, which leads to the formation of periapical lesions, such as granulomas or cysts around the root [34].

Copper hydroxide ions penetrate the root area and enter into the dentin tubules, helping in disintegration and extracellular destruction of the biofilm matrix and destroying the membrane of microbes and spores, fusing the cellular residues and pulp fibrous elements [23]. Cupral’s performance in clinical practice was documented years ago by a histological analysis showing that teeth, endodontically treated and extracted for prosthetic reasons 3 to 6 months later, revealed the presence of copper crystals inside the intra-canalar system, imprinting dentin walls. Moreover, an absence of microorganisms was revealed, ensuring a long lasting endodontic sterility [35]. 

Our data indicated that the Cupral-electrophoresis technique helped stimulate the regeneration of apical destructive focal sites, in infected, obliterated, and curved root canals over 60°. This was likely related to the emersion of Cupral during electrophoresis session into the periapical tissues through the permanent electrical current, thus achieving proteolysis, detaching the apical-cement associated microbial biofilm, which is impossible to achieve using those traditional methods. Accordingly, in vitro, Cupral is demonstrated being efficient against the formation and persistence of bacterial and fungal biofilms [24].

It is relevant to underline, that during clinical Cupral-electrophoresis treatment, the patient does not feel any discomfort or pain. This is because of the very weak current, caused by a low voltage, and only focally used in the endodontic cavity. Upon treatment, high proteolytic activities of hydroxy-cuprate and hydroxyl ions occur in the endodontic cavities. This allows decontamination of the radicular canal and periapical tissue, and in turn will fasten debris absorption from radicular canal, by the organism. Upon such treatment, exudate production (sterile proteolysis) is facilitated in the endodontic environment, decreasing in this manner the pain during chewing process. The Cupral’s high alkalinity (pH = 13) and Atacamit too (pH = 12) does not jeopardize the reinfection of radicular canal, destroying any kind of microbial agent that can enter the endodontic cavity.

To observe specifically the penetration of copper hydroxide, it was decided with the patient’s approval to histologically examine the fractured extracted teeth after 18 months of follow-up. We also agreed on this act, because the body’s electrolyte environment is different from those treated in vitro/ex vivo. This phenomenon was seen quite clearly in the histological sections (see Figure 4 and Figure 5), where a marked impregnation of the internal walls of the root canal by copper sulfite, copper crystals and copper hydroxide was noted along the entire length of the dentinal tubules and their depths. In this way, permanent sterility of the treated teeth and the stimulation of osteogenesis in apical periodontitis can be guaranteed. We did not histologically study the extracted teeth of the control group because it was not in our interest, as Ca(OH)_2_ has been known for many decades and has not sparked interest in our group.

Often, intra- and post-endodontic procedures result in tooth staining and fracture, which is a major aesthetic concern for patients, especially when anterior teeth are involved [36,37,38]. An in vitro study showed that contact with blood does not modify the color alterations in teeth treated with the two novel calcium silicate-based cements after a period of 6 months, where Biodentine showed a higher color stability than MTA [39]. Another in vitro study showed that delayed tooth discoloration was detected for 2 calcium silicate-based cements in a period of one year, but it was more evident for ProRoot MTA than Biodentine [40]. During Cupral treatment on distal teeth, after the end of the second session with electrophoresis, a discoloration of the tooth crown was noted. This is likely due to the diffusion of copper crystals in the endodontic cavity. To avoid this phenomenon as much as possible, the walls of the coronary cavity have to be cleaned carefully. It is recommended, particularly, to avoid this discoloration in frontal teeth, using Cupral combined with Ca(OH)_2_-highly dispersed paste in the ratio of 1:9 [23].

According to the manufacturer, this method has some limitations; it cannot be applied to teeth with vital pulps, such as after vital extirpation, where devitalization must be performed first, nor in patients allergic to copper or other metals. Pregnant women and patients with cardiovascular disease and pacemaker cannot be treated, because of the current.

Taking into account all the above-mentioned points, we can say that (1) the use of Cupral’s solution during the apical periodontitis treatment helped raise the medical success, in both near and distant times. Further, (2) according to [41,42,43,44], the amount of copper, a physiological compound in human body, used during electrophoresis does not show negative or toxic effects. Moreover, (3) the absence of clinical complications and the positive results obtained at the end of this study allowed us to present the Cupral-electrophoresis methodology as a valid alternative for widespread use in various clinical situations, avoiding in this manner the apical traumatic endodontic surgery.

## 5. Conclusions

Our study provides the first evidence on the efficacy of Cupral in the treatment of apical periodontitis. By using both a clinical and radiological 18-month follow-up, we demonstrated the effectiveness of the treatment in the near and distant time. We favor the idea that the action of Cupral and its penetration are indeed optimized through electrophoresis.

The intense periapical tissue regeneration observed paves the way for further in-depth studies to better comprehend Cupral’s clinical impact.

## Figures and Tables

**Table 1:** Outcome after an 18-month survey in the control and Cupral groups.

Control Group				
Age Groups (Years)	No of Treated Teeth	Success (%)	Failure (%)	Extracted Teeth
19–21	11	100	-	-
22–35	16	87.5	12.5	2
36–50	15	86.7	13.3	2
51–65	20	85	15	3
	*n* = 62			
**Cupral Group**				
19–21	8	100	-	-
22–35	22	100	-	-
36–50	30	96.7	3.3	1
51–65	20	90	10	2
	*n* = 80			

## Data Availability

The data presented in this study are available on request from the corresponding author.

## Supplementary Materials

The following are available online at https://www.mdpi.com/1996-1944/14/3/678/s1, Figure S1: An example of how focal size measurement was made using the ruler, before (a) and after (b) treatment in follow-up.
