# Supplementary material for: Copper–Calcium Hydroxide and Permanent Electrophoretic Current for Treatment of Apical Periodontitis
Source: Materials (Basel). 2021 Feb 2;14(3):678. doi: 10.3390/ma14030678 (PMC7867200; doi:10.3390/ma14030678)
Supplement: Supplementary file 1 [file materials-14-00678-s001.pdf]

materials

MDPI

Supplementary Materials

Copper–Calcium Hydroxide and Permanent Electrophoretic Current for Treatment of Apical Periodontitis

Agron Meto [1], Etleva Droboniku [2], Elisabetta Blasi [3], Bruna Colombari [3], Emiljano Tragaj [1], Gabriele Cervino [4], Luca Fiorillo [4,5,*], and Aida Meto [2,3,*]

[1] Department of Therapy, Faculty of Dentistry, University of Aldent, 1000 Tirana, Albania; agronmeto@yahoo.com (A.M.); emiljanotragaj@yahoo.com (E.T.)
[2] Department of Dental Therapy, Faculty of Dental Medicine, University of Medicine, 1005 Tirana, Albania; etleva.droboniku@umed.edu.al
[3] Department of Surgical, Medical, Dental and Morphological Sciences with Interest in Transplant, Oncological and Regenerative Medicine, University of Modena and Reggio Emilia, 41125 Modena, Italy; elisabetta.blasi@unimore.it (E.B.); bruna.colombari@unimore.it (B.C.)
[4] Department of Biomedical and Dental Sciences, Morphological and Functional Images, University of Messina, 98100 Messina, Italy; gcervino@unime.it
[5] Multidisciplinary Department of Medical-Surgical and Odontostomatological Specialties, University of Campania "Luigi Vanvitelli", 80121 Naples, Italy
* Correspondence: lfiorillo@unime.it (L.F.); aidameto@yahoo.com (A.M.); Tel.: +35-569-331-7677 (A.M. & L.F.)

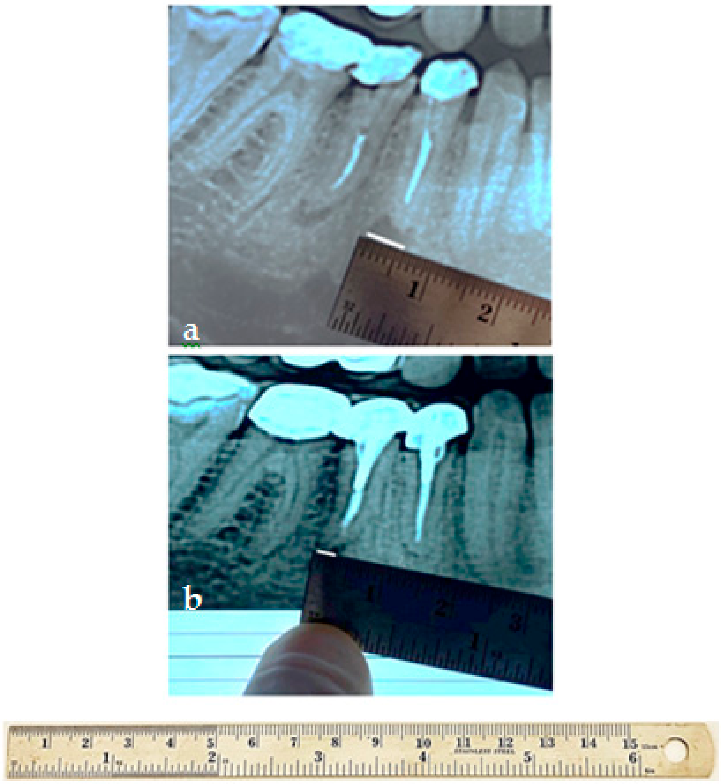

Figure S1. An example of how focal size measurement was made using the ruler, before (**a**) and after (**b**) treatment in follow-up.

Publisher's Note: MDPI stays neutral with regard to jurisdictional claims in published maps and institutional affiliations.

Copyright: © 2021 by the authors. Licensee MDPI, Basel, Switzerland. This article is an open access article distributed under the terms and conditions of the Creative Commons Attribution (CC BY) license (http://creativecommons.org/licenses/by/4.0/).
